# Eco-friendly synthesis of silver nanoparticles from the leaf blades extract of seagrass *Thalassia hemprichii*: Multifunctional agents for biomedical and environmental applications

**DOI:** 10.1016/j.jgeb.2025.100600

**Published:** 2025-10-22

**Authors:** Shibin Eranhottu, Tijo Cherian, R. Mohanraju, N. Sharmila Devi, Jayalakshmi Sanal, Lincy Sara Varghese, P. Ambili, Mini Thomas, Fahmeeda Parveen P.S., Willie J.G.M. Peijnenburg

**Affiliations:** a Department of Ocean Studies and Marine Biology, Port Blair Campus, Port Blair 744112 Andamans, India; b Post Graduate Department of Food and Industrial Microbiology, Department of Botany, Bishop Kurialacherry College for Women, Amalagiri, Kottayam 686561 Kerala, India; c Department of Botany, Bishop Kurialacherry College for Women, Amalagiri, Kottayam 686561 Kerala, India; d Department of Physical Education, Bishop Kurialacherry College for Women, Amalagiri, Kottayam 686561 Kerala, India; e Institute of Environmental Sciences (CML), Leiden University, RA, Leiden 2300, the Netherlands

**Keywords:** Silver nanoparticles, Seagrass, *Thalassia hemprichii*, Dye degradation, Antibacterial

## Abstract

The current study reports for the first time the synthesis of environmentally friendly silver nanoparticles (AgNPs) using leaf blades extract (THE) of seagrass *Thalassia hemprichii*. The AgNPs were described using several spectroscopic methods. The UV–visible spectral study of THE-AgNPs reported absorbance peak at 420 nm. The intrinsic stretching was seen in the FT-IR (Fourier transform infrared) spectrum inferring the presence and role of diverse functional species in the biological synthesis of AgNPs. The AgNPs reported uniform morphology, spherical shape and high stability validated by electron microscopic and zeta potential studies. The biosynthesized silver nanoparticles were employed in experiments including antibacterial, anti-diabetic (α-amylase (77%), α-glucosidase (78%) at 30 μg/ml, antioxidant (85–90%), anti-inflammatory (45–60%) properties and dye degrading properties. The antibacterial efficacy of silver nanoparticles was assessed against pathogenic species of *Staphylococcus aureus* and *Escherichia coli* reporting inhibitory zones of 13 ± 0.3 and 21 ± 0.2 mm, respectively comparable to the standard Gentamycin (22 ± 0.4 mm). The synthesized AgNPs demonstrated excellent dye degradation kinetics in non-photocatalytic dyes methyl orange (96 %; 30 min) and safranin O (95 %; 40 min) along with experimental reusability found to be satisfactorily upto 7 cycles.

## 1 Introduction

Innovations in nanobiotechnology have led to fascinating findings in materials science. This technology now allows for the biosynthesis of environmentally and economically advantageous metal nanoparticles for use in agriculture, food, cosmetics, environmental safety, defense, and healthiness.^1^ Various plant parts, including fruits, leaves, and roots, are commonly employed in biosynthesis, which are also referred to as “green synthesis”.^2^, ^3^ Nanomaterials can also be produced by conventional physical and chemical processes. But whereas physical procedures need a lot of energy, chemical ones have disadvantages like biological toxicity in humans and environment. Moreover, the proper shape- size- distribution, crystallinity, composition, and morphology are absent from physically produced nanoparticles.^4^ The catalysis and microbiological sectors have employed assortment of metallic- and −derived oxide nanoscale schemes. Among the diverse kinds of NPs, silver nanoparticles attracted unique significance owed to their special optoelectronic and physiochemical distinctiveness popularized them as an efficient catalytic, anticancer, bactericidal, and fungicidal agent. AgNPs are highly valued due to their ability to withstand antibiotic resistance against microorganisms that are resistant to many drugs. AgNPs exhibit excellent antibacterial potential against different pathogenic organisms, such as *Escherichia coli* and *Staphylococcus aureus*.^5^ Silver nanoparticles (AgNPs) are remarkable owing to their high surface-to-volume ratio, narrow plasmon resonance, special physicochemical properties, and wider applications in biology, microelectronics, and medical research. Unlike other metal NPs, AgNPs have garnered immense consideration owing to their extensive usage in a variety of pharmacologically and economically significant goods. The traditional synthesis methods— chemical, hydrothermal, thermal, and physical modes—are costly, dangerous, dependent on hazardous materials. Therefore, the primary focus is highlighted on green synthetic techniques that use biological resources to create NPs effectively. The synthetic production of sustainable nanoparticles employing renewable and environmentally safe chemicals as reducing and capping agents is the basis of this eco-friendly technique.^2^

Silver based nanoparticles have attracted the focus of much research because of their distinctive characteristics and possible use in a range of fields, including medicine.^6^ The impact of silver oxide nanoparticles on the body's ability to reduce inflammation is one of these research topics. Inflammation is a physiological reaction triggered by the immune system of the body to protect against pathogens, injured tissues, or allergens.^7^ Cell damage is another reason why inflammation might happen. Conversely, chronic or severe inflammation is related with certain maladies, such as cancer, heart disease, arthritis, and can be dangerous. Effectively managing inflammation is therefore a key goal in the therapeutic management of numerous medical conditions. Cells generate hydroxyl radical, superoxide anion, singlet oxygen, and other extremely dangerous reactive oxygen species (ROS) during regular metabolic processes. However, ROS can harm macromolecules like DNA, proteins, and lipids when present in excess. ROS is necessary for some biological processes, including signal transduction and energy production in driving biological progression.^8^ Researchers have reported a link between the pathophysiology of oxidative diseases such as diabetes, cardiovascular problems, cancer, and oxidative damage. Research has demonstrated the important role antioxidants play in preventing a wide range of ailments. By delaying or postponing an oxidative chain reaction's initiation or propagation stages, antioxidants can stop ROS-mediated damage. By the production of ROS (reactive oxygen species), AgNPs deactivates mitochondrial respiratory mechanisms. In this manner, they can also cause damage to the DNA of cancer cells and stop them from proliferating by triggering apoptosis.^9^ Around the globe, cancer is thought to be accountable for one in every six fatalities. It is commonly known that 90–95% of malignancies are prompted by gene alterations brought on by environmental causes. AgNPs have gained more attention lately, mainly because of their anticancer,^10^ antioxidant,^11^ and antibacterial^12^ properties. Furthermore, the increasing antimicrobial resistance as a result of overuse or improper administration is seen as a portent of the end for currently available antibiotics that are extensively utilized globally. Silver nanoparticles, as alternative activity pathway, interact with the cellular surface of bacteria leading to their rupture, offering them as a viable substitute for antibiotics.^13^

Globally, 422 million individuals suffer from diabetes; the majority of them live in nations with middle to low incomes.^14^ Additionally, 1.6 million people die from diabetes worldwide each year. The recent years have shown that nanotechnology can be very helpful in both the detection and management of diabetes. Medicinal herbs have long been used to cure a wide range of illnesses. Due to their affordability and environmental friendliness, plant extracts have gained immense attention in past years when used in the fabrication of AgNPs. Some bioactive compounds found in medicinal plants, like flavonoids, antioxidants, and phenolic molecules, are acknowledged as the imperative sources in the creation of drugs to treat diabetes mellitus Type II. The two primary enzymes involved in the metabolism of carbohydrates are α-amylase and α-glucosidase, which breakdowns carbohydrates into monosaccharides and spiking up the blood sugar levels.^15^ Therefore, one of the most crucial methods for treating diabetes is the inhibition of these enzymes. On the other hand, Alzheimer's disease (AD) is a brain condition that progresses fatally and has emerged as a significant threat to public health, especially in mechanized nationalities with higher standards of living. It is a prevalent type of dementia, especially in elderly people; distinguished by aberrant behavioral changes and irreversible neuronal loss. Treatments for AD include disease-modifying therapy, psychosocial interventions, psychiatric medications specifically inhibitors of cholinesterase [that block the breakdown of neurotransmitters, AChE (acetylcholine) and BuChE (butyrylcholine)]. Many studies have been conducted that show the prospective synthetic metallic nanoparticles derived from plants as anti-cholinesterase medications.^16^ Numerous investigations have demonstrated the potential of metal nanoparticles produced from plants as anti-cholinesterase drugs.^16^, ^17^, ^18^

Noble-metal nanomaterials have remarkable chemical and physical qualities that enable unique interactions with the environment. These properties include a bottom-up approach, synthetic plasticity, discrete morphologies, and low cost.^19^ Using AgNPs as plasmonic sensors effectively targets heavy metals and organic elements found in water, enhancing the environmental functions of photocatalysts that promote the oxidation and destruction of dyes and pesticides.^20^ The environmental impact of manmade nanomaterials is a topic of increasing interest these days.^21^ The topic of enduring the toxic effects of AgNPs in fluid settings has been the focus of several literatures.^19^ The degrees of silver nanoparticle toxicity vary depending on the cumulative amount of exposure, and the ecosystem of organisms that exists in the environment determines the highest levels of toxicity in each taxon.^22^ In addition to wastewater treatment, a wide range of industries use metallic AgNPs for their exceptional beneficial features. These industries include biomedicine, cosmetics, drug therapy, culinary goods, DNA sequencing, coating, biology, and other sectors.^23^ Nonetheless, a large portion of AgNP research is concerned with wastewater treatment, water purification, and dye removal.^24^ The synthesis techniques for AgNPs rely on variations in reaction conditions and reactants during the process, and can be based on repeatability and cost-effectiveness.

Seagrasses are encased marine angiosperms that, except in the Polar areas, are found in all tidal and subtidal zones of the oceans.^25^ They flourish swiftly and produce copious organic matter, which gives rise to a multitude of secondary metabolites with different structural characteristics.^26^ Seagrass possesses phenolic compounds, specifically flavonoids and polyphenols, which enhance its antioxidative and pharmacological properties.^27^ Thus, the plant may include compounds with anticancer (ferulic acid, cinnamic acid), anti-HIV, immunostimulants (chicoric acid, caffeic acid), antibacterial, and antioxidant properties.^28^ Reductase and other bioactive metabolites such as alkaloids, terpenes, phenols, polyphenolic, ascorbic acid, citric acid, and flavonoids present in seagrass extracts may function as reducers.^29^ The synthesis of seagrass-mediated AgNPs and their various uses are not well studied; as most researches focus on microbial- and plant-mediated AgNPs synthesis and its various uses, but little attention is paid to the unique potential of seagrasses. Nonetheless, as seagrass has significant antioxidant and reducing qualities, there is increasing interest in using its plentiful waste to extract bioactive chemicals and synthesize AgNPs with a wide range of industrial, environmental, and therapeutic uses. a) Limited taxonomic diversity: There aren't many seagrass species that have been utilized. Worldwide, many seagrass taxa and species are still unexplored. This restricts knowledge of the range of synthesis potential, attributes of nanoparticles, and prospective applications; b) Limited control over size, shape, and stability: While characterizations (UV–Vis, TEM, XRD, FTIR, etc.) are common, less effort is put into adjusting the size, shape, and dispersion of nanoparticles by synthesis parameter modifications. Systematic research on long-term stability (shelf-life; across multiple environments) is rare; c) Mechanistic comprehension is limited: The precise biomolecules (such as proteins and polyphenols) in seagrass extracts that stabilize AgNPs and reduce Ag^+^ ions are frequently only partially discovered; d) Limited spectrum of potential uses beyond biological assays: Applications for seagrass-derived AgNPs, such as environmental remediation (e.g., water treatment, pollutant degradation), sensors, catalysis, etc., are either rarely or never investigated. They are rarely used, for instance, in material science (coatings, composites), pollutant treatment, or colour degradation. Keeping in the view, the introduction of extract from the leaf blades of seagrass *Thalassia hemprichii* in the biological production of AgNPs is the originality of this work and serves as “first time report” explicitly synthesizing metal nanoparticles. The seagrass extract (THE) was employed in the AgNPs synthesis; characterized by the techniques: UV–Visible spectroscopy, TEM, SEM, zeta potential, FTIR, TGA, DLS and explored for bioactivities such as antibacterial activity, antioxidant, anti-diabetic, anti-inflammatory and dye removal assays. In the present work, process of catalysis was used in the catalytic activity to break down certain harmful dyes. Furthermore, no research has yet to be done on manufacturing THE-stabilized AgNPs or the rate at which they degrade in response to different dye classes. The approach used for this was economical, environmentally responsible, and sustainable and the impact of artificially generated AgNPs on deterioration across various time intervals was investigated.

## 2 Materials and methods

### 2.1 Collection and processing of plant material

Seagrass *Thalassia hemprichii* was collected with sterile scissors and forceps from the intertidal region of Burmanallah (11^°^52′21′′N, 92^°^69′12′′E), South Andaman, at low-tide in sterile polythene bags and were transported to the laboratory under sterile conditions. The seagrass sample was identified based on the morphological characters.^30^ The leaf blades were washed thrice with filtered and autoclaved seawater and later with autoclaved distilled water to ensure that no loosely bound settlements, such as soil or other debris, were present on the leaf blades. Following washing, the leaf blades samples were shade dried and grounded into fine powder. In the preparation of aqueous extract (THE), 50 g powdered *T. hemprichii* was added to 100 ml sterile distilled water, thoroughly mixed, and heated for 30 min at 50°C under continuous stirring. The solution was filtered by using Whatman’s filter paper and stored at 4°C until further analysis.

### 2.2 Biochemical and phytochemical analyses of seagrass

The biochemical indices such as protein, lipid, and carbohydrate of seagrass were estimated based on standard laboratory techniques. Also, the phytochemical examination was carried out for the analysis of organic compounds. All the analyses were carried out using the earlier techniques,^31^, ^32^, ^33^, ^34^ albeit with a few minor adjustments (descriptions of the techniques are mentioned in the supplementary file S1).

### 2.3 Synthesis of biosynthesized AgNPs

To synthesize silver nanoparticles, a fixed volume of seagrass extract was used. For example, 5 ml THE was combined with 45 ml aqueous AgNO_3_ (1 mM) in a 150 ml conical flask, and the mixture was kept at 120 rpm for 24 h under incubation at room temperature. A change in color from greenish to brownish black indicates the production of silver nanoparticles. The sample was then kept at 4°C until further examination.^35^

### 2.4 Morphological and physico-chemical characterization

The vibrational, structural, and morphological characteristics of silver nanoparticles were investigated using characterization techniques of electron microscopic examinations (TEM, SEM), UV–visible spectroscopy, Fourier transform infrared (FT-IR) spectroscopy, zeta potential, DLS, TGA. The operational specifications were followed as mentioned in the studies of Cherian *et al*.^36^ and Ali^37^.

### 2.5 Biological activities of CCLE-AgNPs

The biological activities of THE-AgNPs: anti-diabetic (inhibition of enzymes α-amylase and α‑glucosidase),^38^, ^39^ antioxidant (FRSA, TRP, TAC, ABTS),^40^ anti-inflammation (COX-1, COX-2, 15-LOX, sPLA2),^41^ anti-bacterial, anti-biofilm and dye degradation^42^ were ascertained. The detailed information of assays is mentioned in Supplementary file S1.

### 2.6 Statistical analysis

The experimental values were reported as mean ± standard deviation calculated by one-way ANOVA followed by Tukey's post-hoc test for each experiment run in triplicates. The p-value (p ≤ 0.05) was considered as level of significance.

## 3 Results and discussion

The aqueous extract of *T*. *hemprichii* was subjected to phytochemical examination in order to detect the presence of organic compounds. The findings of the aqueous extract of *T*. *hemprichii* revealed a number of chemical components, each of which is described in detail in Table 1.

**Table 1:** Phytoconstituents in *T*. *hemprichii* aqueous extract (+presence, − absence).

S.No	**Compound**	**Observed result**
1.	Glycosides	+
2.	Flavonoids	+
3.	Alkaloids	+
4.	Flavone glycosides	+
5.	Phenol	+
6.	Sulphates	+
7.	Sterols	+
8.	Ketones	+
9.	Fatty acids	−

### 3.1 Mechanism of AgNPs synthesis

**Fig. 1:**
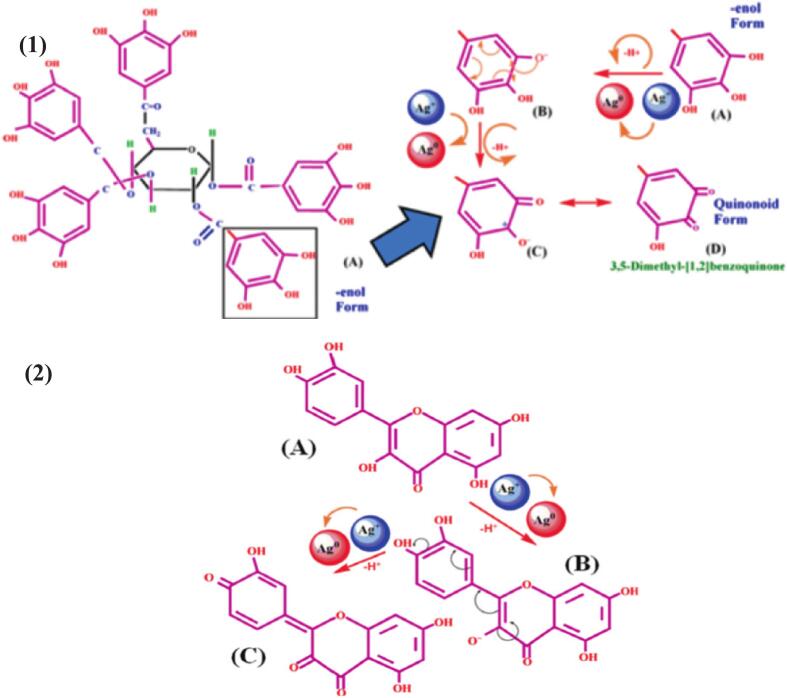
General mechanism of AgNPs synthesis mediated by phytocompounds present in plant extracts.

It is no accident that biological processes for synthesizing AgNPs continue to attract attention. There are as many different biological living organisms— plants, algae, fungi, and bacteria— that can synthesize AgNPs as there are biological matrices for their synthesis. The availability of the agents required for synthesis, low toxicity, high productivity, and energy efficiency are the aspects that favour the use of biological agents for synthesis. The goal of this ecologically benign, biocompatible process is to synthesize AgNPs with potentially beneficial qualities for use in medicine. Silver nitrate (AgNO_3_) interacts with biological extracts (plant, fungal, or bacterial) chemicals in the “bio-factories” method of synthesizing AgNPs. AgNPs were typically synthesized using chemical and physical processes but constrained with numerous disadvantages. Physical techniques demand costly equipment and a lot of energy. However, the primary drawbacks of the chemical procedures include their employment of very hazardous chemicals, contamination of precursors, environmental pollution, and carcinogenic solvents. The direct involvement of different phytocompounds (tannins, terpenoids, flavonoids, etc.) and biocomponents (enzymes, proteins) in the reduction and stability of nanoparticles is a significant distinction between biological and conventional (physical and chemical) techniques. Three steps are involved in the formation of silver nanoparticles: first, biological catalysts in a “factory” of synthesis (here THE) reduce Ag^+^ to Ag^0^; second, oligomeric clusters agglomerate and stabilize to form colloidal silver particles; and third, AgNPs are formed.^43^, ^44^ (Fig. 1). Biomolecules help reduce Ag^+^ ions to elemental silver (Ag^0^) by donating electrons from functional groups such as carboxyl (–COOH) and hydroxyl (–OH).^44^ Particle synthesis induces the medium's black hue to gradually but sharply increase along with SPR. The formation of the nanoparticles is indicated by the ensuing solution's colour changing from pale yellow to brown/ brownish black.^44^ Then, reduced silver atoms start to form aggregates, which can be kept to a minimum if the reaction medium contains a capping agent; if not, smaller aggregates keep growing and eventually form bigger aggregates. AgNPs are synthesized when capping agents contact with Ag^0^ by covalent bond formation, electrostatic interactions, or van der Waals forces.

(Adapted from Parashar et al.)^47^

Many biological substances may be able to contribute to the production of AgNPs, which means that plants are a limitless supply of nanoparticles with a wide range of characteristics. The biomolecules in plant extracts interact with AgNO_3_ in the straightforward process of plant biosynthesis. Silver ions (Ag^+^) are reduced to Ag^0^ and stabilized when hydroxyl groups (–OH) are present in plant biomolecules.^45^ Flavonoids and tannins are examples of phenolic chemicals that can act as reducing agents. Because the aromatic rings of these phenolic compounds are so nucleophilic, it has also been proposed that they exhibit chelating properties. These biomolecules' carboxyl (–COOH) and –OH functional groups are essential to the reduction process. Electrostatic interactions are established between the positively charged Ag^+^ ions and the negatively charged COO− in organic acids or O− in phenols. Through tautomeric transitions, flavonoids' diverse functional groups have an improved capacity to decrease metal ions by generating reactive hydrogen atoms. The keto form of the flavonoid participates in the synthesis where it changes into the enol form upon the reactive hydrogen release; however, the enol form was unstable and reverted to the keto form given that it contained two –OH groups on the same carbon. Consequently, Ag^+^ is changed into Ag^0^ by the released reactive hydrogen, and these two elements then combine to synthesize AgNPs.^46^

**Fig. 2:**
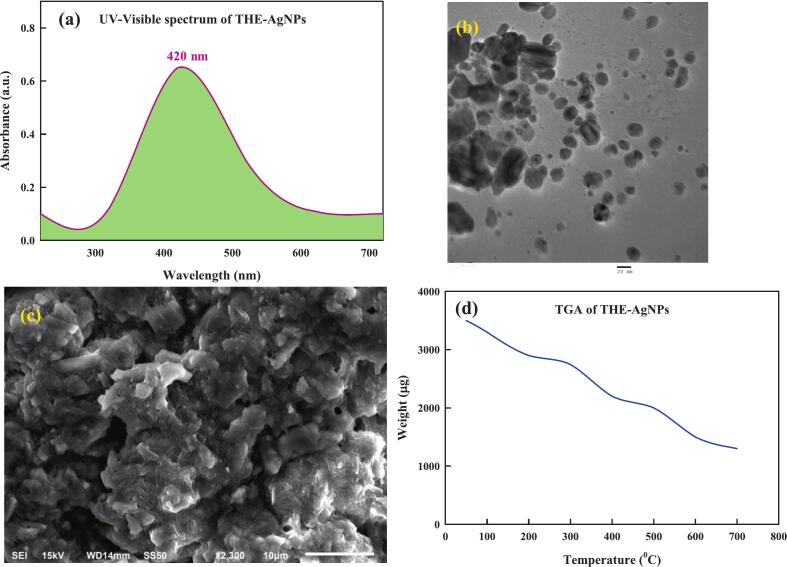
Characterization of THE-AgNPs (a) UV–visible spectrum; (b) TEM; (c) SEM; (d) TGA.

UV–visible spectroscopy is a fundamental and practical method for the thorough classification of generated nanoparticles. It is also employed to investigate the consistency and production process of nanomaterials.^48^ AgNPs exhibit significant optical properties that cause them to interact strongly with particular wavelengths of light.^49^ Additionally, UV–Vis spectroscopic analyses are quick, straightforward, and practical. It measures several NP types swiftly and is robust and discriminating in nature. Moreover, calibration is not necessary for the characterization of colloidal suspension particles.^50^ Electrons can effortlessly flow between the conduction- and valence- band of silver nanoparticles since they are very near with each another. Silver nanoparticle electrons get collaboratively into oscillations in synchrony with the light waves due to free electrons, forming an SPR absorption band.^51^ SPR is a well-known phenomenon that causes noble metals to display unique optical properties. The current investigation used the solution's UV–vis spectra to determine that the extract of *T. hemprichii* as the catalyst that changed AgNO_3_ into silver nanoparticles. The addition of seagrass extract to clear and colorless aqueous solution of AgNO_3_ resulted in the coloration of resulting solution to light brownish hue which intensified to brownish black as the reaction progressed with respect to time. The chemical constitution of flavonoid and phenols chemicals in the seagrass extract led to the shift in hues to silver oxide (AgO) and a corresponding decrease in Ag^+^.^26^, ^52^ Moreover, the biomolecules' increased extract concentrations serve as reducers that cap the surfaces of the nanoparticles and inhibit their aggregation. Similar studies reported that the diverse extract components cause the formation of symmetrical nanoparticles.^53^, ^54^ The primary reason for the dark-brown color observed in the solution is the activation of surface photoluminescence (SPR) in AgNPs. At λ = 420 nm, an SPR band with a good definition for silver nanoparticles was observed (Fig. 2a). Similar reports of Dilipan *et al*.,^28^ Fenfen *et al*.,^53^ and Ibrahim *et al*.^54^ inferred that the seagrass-mediated AgNPs had a dark brown color and absorbance peak at 400–420 nm. Furthermore, in some instances peak widening is observed which is suggestive of the spherical and polydispersed nature of the particles highlighted by an increase in the absorption peak precisely corresponding to the duplication of AgNPs.^55^ Also, the shape, size, and metal-dielectric constant of the metal nanoparticle concerning the immediate medium effects on frequency and amplitude of SP absorption.^56^

**Fig. 3:**
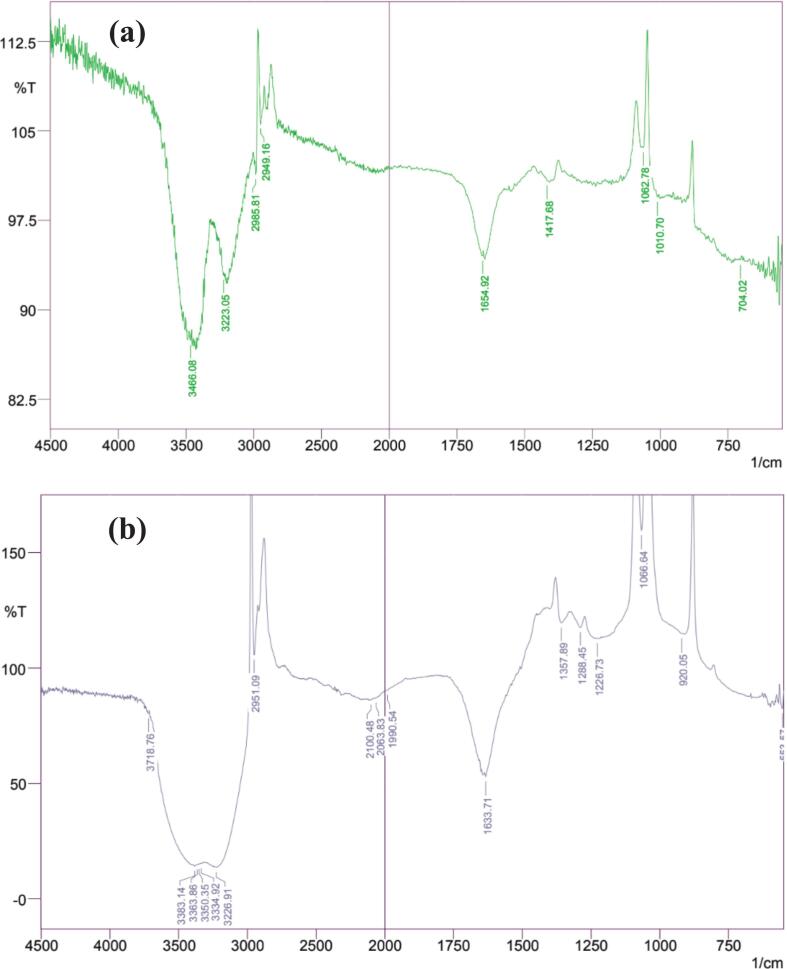
FTIR of (a) THE (b) THE-AgNPs.

The surfacial morphology of THE-AgNPs was reported by employing electron microexaminations (Fig. 2b, c). The micrographs exhibited sphere-shaped nanoparticles with regular grain boundaries facilitated by nanosheets and high degrees of homogeneity with no aggregation attributable to the presence of phyto-derived chemicals acting as stabilizers in THE.^57^ Also, it was demonstrated that solely the seagrass extract constituted the forefront of the reduction as the capping reagent was used in the stabilization of AgNPs as the latter had no interaction even in the gathered aggregates.^58^ The TEM examinations typified the average particle size of silver nanoparticles as 13.4–21.32 nm. The particle characterization, including determining the surface charge and size of synthesized silver nanoparticles, can be accomplished by the use of zeta potential and DLS measurements. The hydrodynamic size distribution of silver nanoparticles was measured by DLS (not shown). The average size of silver nanoparticles was found to be 25.4 nm. The colloidal stability of silver nanoparticles was tested using zeta potential measurements (not shown), whereas the recorded value of − 21.45 mV indicates a higher stability. A kind of AgNPs associated electric potential that forms a double electrical layer and rises out of the solution is called the zeta potential (ZP). The stability and surface charge of AgNPs are usually assessed using ZP.^59^ The NPs ZP is regarded as neutral when it falls between −10 and + 10 mV. Strongly cationic and anionic AgNPs are defined as having a ZP > +30 mV and < -30 mV, respectively. According to reports, the least noxious AgNPs are negatively charged, whereas the most hazardous AgNPs are positively charged.^60^, ^61^ ZP values above this range imply aggregation, sedimentation, and flocculation, whereas ZP values between −30 mV and + 30 mV generally indicate high stability of AgNPs.^62^ The functional groups of various biochemical compounds present in plant extracts caps the AgNPs and are primarily responsible for conferring negative surface charge.^45^ A lower electrostatic barrier is indicated by the − 21.45 mV surface charge, which improves the interaction between the synthesized AgNPs and biological cells which is critically indispensable for the utilization of THE-AgNPs in biological activities. The mean dimension of AgNPs determined using DLS was found to be slightly greater than that of the electron microscopic analysis due to the development of hydrodynamic spheres surrounding particles in water-based environments, leading to a more corpulent determination of the diameter of the particles when examining the scattered light under DLS quantification.^58^, ^63^ The technique of TGA was used to examine the temperature behavior of THE assisted AgNPs. It is a useful analytical method for tracking how a sample's weight varies over time as its temperature changes as it is heated in a controlled environment. Under a nitrogen atmosphere, the THE-AgNPs thermogram showed three distinct stages of degradation at temperatures 20°C-600°C at 10°C/minute (Fig. 2d.). The desorption of surface-adsorbed moisture and volatile organic molecules may be the cause of first stage weight loss (0.321 mg, 4 %), which happened at < 200°C. The biological organic compounds of carbohydrates, phenolic acids, and flavonoids that were trapped on the THE-silver nanoparticles surface decomposed, resulting in second weight loss (3.01 mg, 25.89 %), which occurred between 200°C-450°C.^64^ Between 450°C and 600°C, a consistent weight loss (0.501 mg, 5.40 %) was observed in the third stage. This weight loss is most likely related to the thermal breakdown of oxygen molecules resistant and aromatic compounds present on THE-AgNPs.^65^ TGA showed that volatile and bioactive organic compounds present in THE bound to the surface of the resultant nanoparticles, which showed that the total breakdown of THE-AgNPs owing to the loss of bioactive molecules. The AgNPs synthesized using *S*. *multicaulis* stem extract exhibited a comparable thermal behavior to green produced AgNPs.^64^ The extract of *T. hemprichii* was used to synthesize AgNPs, and FTIR analysis evaluations were performed to determine the prospective biological molecules that are accountable in the reduction of silver ions and the crowning of silver nanoparticles. The results revealed the presence of various functional groups, including saponins, tannins, flavonoids, and alkaloids (Fig. 3, Table 2). The multiple functional groups, like CH, –OH, C=O, –NH groups, were detected in the FTIR, indicating that the seagrass extract AgNPs contains flavonoids and polyphenols that had been substituted with amine and hydroxyl groups. A slight alteration in peak amplitude was noted, which could be related to the variation in capping molecules and the kind of coordination between AgNPs and the metal surface.^42^ Plant extracts have low synthetic process, excellent productivity, and minimal toxicities, and are preferred over microbial nanoparticle production. AgNPs are most likely synthesized from plant extract by an enzymatic method similar to that which microorganisms employ. The chemicals employed in the final capping and stabilization of NPs must be different from those used for microbes as cellular machinery of plant have a composite of different anti-oxidative metabolites that prevent oxidation and harm to biological structures. This indicates that biomolecules, such as saponins, enzymes, and glycosides, can aid in the stabilization of the nanoparticles. The studies indicates that the –COOH and –OH groups in silver ions attach to water-soluble compounds when metallic salts are introduced to a plant extract.^58^

**Table 2:** FT-IR result of aqueous extract of *Thalassia hemprichii* (THE) and AgNPs.

**Wavelength (cm^−1^)**	**Appearance**	**Functional group**	**References**
**THE**			
3466–3223	Strong	O–H stretching	42
2985–2949	Low	C–H stretching	42,58
1654–1417	Medium	C=C stretching	36
1062–1010	Medium	C–O stretching	58
704	Low	C–H bending	36
**THE-AgNPs**			
3718–3226	Strong, broad	O–H stretching	36
2951	Strong, narrow	N–H stretching	36
2100–1990	Low	C=C stretching	42
1633–1357	Strong	C=O stretching	58
1288–1226	Low, narrow	C–O stretching	36,42
1066–920	Strong, broad	CO–O–CO stretching, C–H bending	36

**Fig. 4:**
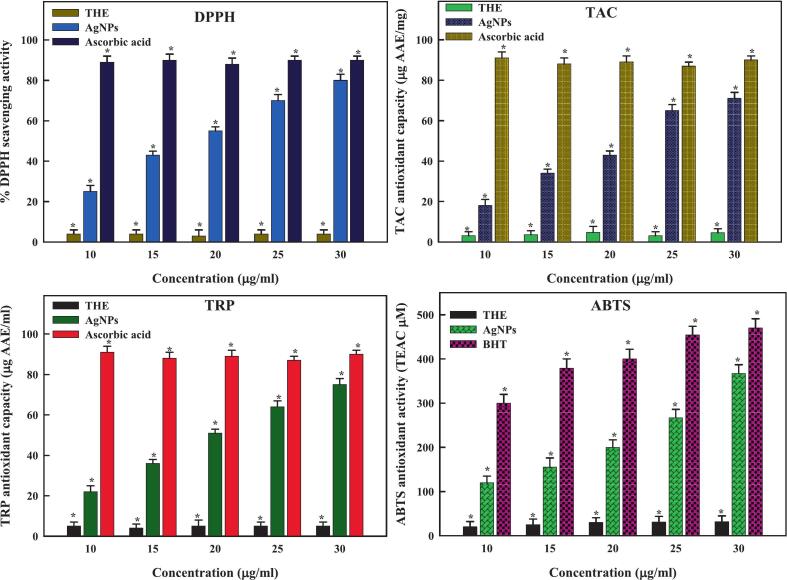
Antioxidant activity of THE-AgNPs.

The phytochemical examination of leaf blade extract of *T*. *hemprichii* demonstrated the role of biological organic species in the biosynthesis of stable nanoparticles (NPs). The biofunctionalized NPs exhibit increased biological activity due to the presence of organic species that may have therapeutic qualities.^66^ There is a widespread perception that nanoparticles made from biomaterial generated from plants or a particular plant extract will probably have bioactivities that are comparable to those of the plant extract.^67^ In general, plant extract-derived nanoparticles have greater or superior biological functionality than plant extracts.^68^ Table 3 enlisting synthesis and biological activities of marine plant-based nanoparticles has been listed below. The *T*. *hemprichii* plant extract contains a wide range of derivatives that contain oxygen, including proteins, sugars, enzymes, polyphenols, and flavonoids. Given this, it presents a promising avenue for producing extracellular metal nanoparticles from metal salts by serving as an exceptional supplier of reducers and stabilizers. The DPPH (or FRSA) technique was utilized to quantify the antioxidant capacity of extract (THE) and biosynthesized silver nanoparticles, with ascorbic acid serving as a standard control. Fig. 4 illustrate the effects of variable dosages of green-produced AgNPs on DPPH free radical inhibition. Our findings show that THE-AgNPs work exceptionally well as free radical scavengers and boosted their DPPH activity in a dose-dependent manner. At 10–30 µg/ml, green-produced THE-AgNPs demonstrated scavenging capacity with a mean IC_50_ value (16.02 ± 0.03 µg/ml; Table 4). A lower IC50 indicated higher potency along with a notable difference observed in comparing the antioxidant activity to traditional ascorbic acid. The extract THE-derived silver nanoparticles exhibited greater antioxidant capacity than THE; the variance may have resulted from the samples used in the study having varied chemical constitution.^69^ Similar reports were also reported by Hanachi *et al*.^70^; Gharari *et al*..^64^, ^71^, ^72^ The composite of numerous antioxidant metabolites in plant cells, which protect biological components, including phenolic and flavonoid substances, from oxidation and damage, may be linked to AgNPs' capacity as antioxidants. As a result of the Ag ions' physicochemical interactions with THE's functional groups, these compounds encapsulate AgNPs, imparting greater surface area and spherical shape.^73^

**Table 3:** Comparative account of marine plant-based nanoparticles.

**Plant species**	**Nanoparticles**	**Concentration (μg/ml)**	**Activities**	**References**
*Cymodocea serrulata*	TiO_2_	36.42–68.85 μg/ml	Antibacterial, Antibiofilm, Antioxidant	^74^
	Ag		Anticancer, Antioxidant, Antiglycemic	^28^
*Cymodocea serrulata*	CuO		Antibacterial	^75^
*Halophila decipiens*	Ag	25–100 μg/ml	Antioxidant	^76^
*Cymodocea rotundata*	Ag	20–125 μg/ml	Antioxidant, Anti-inflammatory	^77^
*Posidonia oceanica*	chitosan-selenium nanocomposite	25 μg/ml	Antimicrobial, Antioxidant	^78^
*Champia parvula*	Ag	100 μg/ml	Antioxidant, Antimicrobial, Anticancer	^79^
*Sargassum polycystum*	Ag	100 μg/ml	Antibacterial, Anti-mycobacterial	^80^
*Sargassum*	ZnO		Antibacterial, Anti-Inflammatory	^81^
*Stoechospermum marginatum*	ZnO	46.88 μg/ml	Anti-Dengue	^82^
*Avrainvillea amadelpha*	Ag	6.3–100 ppm	Activity against second larval instars of the housefly *Musca domestica*	^83^
*Codium edule*	Ag	6.3–100 ppm	Activity against second larval instars of the housefly *Musca domestica*	^83^
*Polycladia crinita*	Se	25 and 50 mg/kg	Antioxidant, Anti-inflammatory	^84^
*Caulerpa taxifolia*	Ag	40 µg/ml	Anticancer	^85^

**Table 4:** IC_50_ values of THE, AgNPs, and ascorbic acid.

	**THE**	**AgNPs**	**Ascorbic acid**
**IC_50_ (µg/ml)**	85.03 ± 0.08	16.02 ± 0.03	11.54 ± 0.07

**Fig. 5:**
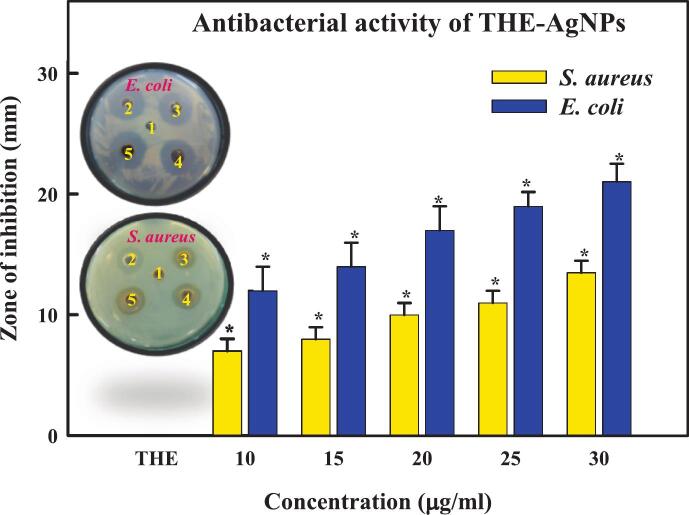
Antibacterial activity of THE-AgNPs (1 = THE, 2 = 10 µg/ml, 3 = 20 µg/ml, 4 = 25 µg/ml, 5 = 30 µg/ml).

**Fig. 6:**
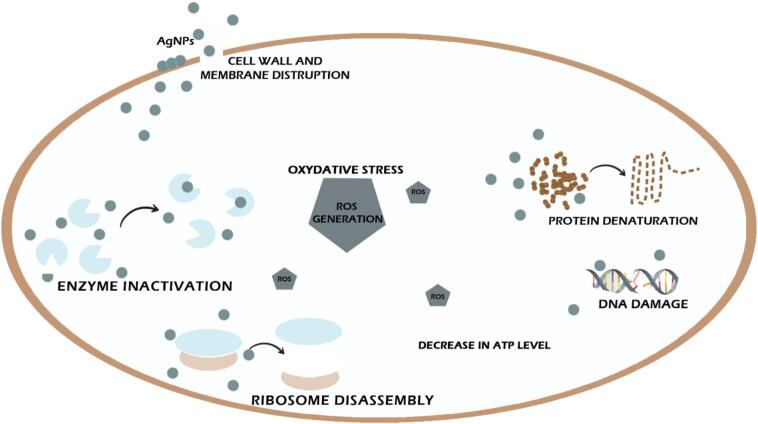
Mechanism of antibacterial activity by AgNPs.

In the current investigation, Table 5 and Fig. 5 displays the antibacterial activity results of inhibitory zones caused by THE, positive control (Gentamycin), and THE-AgNPs. The antibacterial action of THE-AgNPs was found to be greater than that of THE extract, with values for *S*. *aureus* (13 ± 0.3 mm) and *E. coli* (21 ± 0.2 mm). The types of microbes, dose, pH, temperature, colloidal state, shape, size, and zeta potential are some of the variables that affect the antibacterial potential of silver nanoparticles.^64^ While the mechanism fundamental to the antibacterial activity of silver nanoparticles is unknown, it has been suggested that AgNPs may continuously generate Ag^+^ ions, which are supposed as one of the means of destroying microorganisms^86^ (Fig. 6). Ag^+^ ions can adhere to the cytoplasmic membrane and cell wall due to their affinity for sulfurous proteins and electrostatic attraction. The adhered Ag^+^ ions can augment the permeability of cytoplasmic membrane, which can cause the rupture of bacterial envelope.^87^ The absorption of unbound Ag^+^ ions into the cells can inactivate the respiratory enzymes, which stops the synthesis of adenosine triphosphate and produces reactive oxygen species (ROS). ROS can be the primary agent responsible for disrupting cell membranes and modifying DNA (deoxyribonucleic acid). As sulfur and phosphorus are necessary constituents of DNA, the interactive dynamics between these constituents and Ag^+^ ions can cause complications with cell division, replication of DNA, or even the death of the microorganisms. Ag^+^ ions can also stop protein synthesis by causing the denaturation of cytoplasmic ribosomes.^88^ The change in membrane permeability and creation of pits in the outer membrane due to the gradual release of membrane proteins and LPS (lipopolysaccharide) molecules are two other hypothesized mechanisms of AgNP-induced membrane degradation in bacteria like *E*. *coli*. Bacterial cells are unable to appropriately regulate transport through the plasma membrane when their membranes deform, which leads to a marked increase in permeability and ultimately cell death. The closely packed LPS molecules that make up the majority of the outer membrane of *E*. *coli* cells are known to function as an efficient permeability barrier.^89^ Similar to how silver interacts with the thiol groups of transport proteins and respiratory chain proteins to disrupt their normal function, it has also been suggested that the sites of interaction between AgNPs and membrane cells may be caused by sulphur comprising proteins. The metabolism of breathing organisms naturally produces reactive oxygen species (ROS).^90^ Highly reactive radicals are produced when ROS generation is induced, and these radicals kill cells. A failure in membrane function can result from an excess of ROS damaging membrane lipids. The cellular donor ligands that coordinate iron may be interfered with by some transition metals. An increasing amount of evidence indicates that the solvent-exposed [4Fe-4S] protein clusters are the main targets for different metals. When metals destroy [4Fe-4S] clusters directly or indirectly, more Fenton-active Fe may be released into the cytoplasm, which would enhance the production of ROS. The fact that certain Fenton-inactive metals (like Ga, Hg, and Ag,) produce ROS and that cells need or intensify ROS-detoxification enzymes to tolerate harmful concentrations of these nanoparticles may be explained by the capacity to trigger the release of iron from these proteins as well as from other iron-based proteins.^91^ AgNPs exposure causes morphological changes in bacterial cells, including cytoplasm shrinkage, cell wall membrane disintegration, condensation and localization of DNA in the centre of the cell, and cell membrane deterioration that permits leak of intracellular contents.^92^ Alongside morphological changes, bacterial cells undergo physiological modifications as well. They go into a proactive but uncultured stage where their physiologic quantities can be monitored but they are unable to proliferate and divide.^93^ Novel compounds with synergism, primarily natural chemicals combined with nanoparticles, have developed new biological applications, including cancer medications, labeling reagents, bio-imaging devices, medication delivery systems, antimicrobial substances, diagnostics, therapeutic drugs, etc.^94^ Plant based extracts are preferential over microbial produced NPs due to their excellent viability, high efficiency, speed, and low synthesis rate. A combination of different antioxidant metabolites found in phyto-cells, which guard against oxidation and harm to constituent parts of life, is probably what drives the enzymatic synthesis of AgNPs from plant extracts.

**Table 5:** Antibacterial activity of green synthesized THE-AgNPs.

**Bacteria**	**THE**	**AgNPs (µg/ml)**					**Gentamycin**
		**10**	**15**	**20**	**25**	**30**	**30 mcg**
*E. coli*	0	17 ± 0.5	18 ± 0.4	19 ± 0.6	20 ± 0.1	21 ± 0.2	22 ± 0.4
*S. aureus*	0	11 ± 0.3	11 ± 0.3	12 ± 0.5	12 ± 0.2	13 ± 0.3	15 ± 0.5

**Fig. 7:**
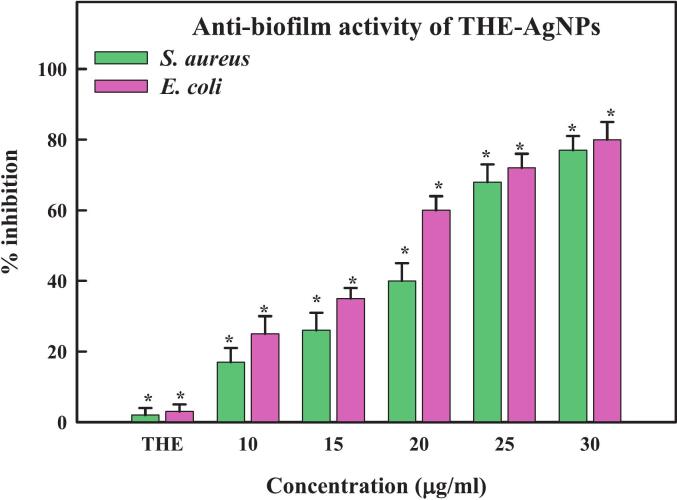
Anti-biofilm activity of THE-AgNPs.

Bacteria may form biofilm during an illness to shield themselves from hazardous environments.^95^ Therefore, if a specific substance is used over a long period bactericidal medication and cannot break through the biofilm that the bacteria have developed, its efficiency may decrease.^96^ These occurrences have increased in frequency recently, becoming a significant health concern. Since Ag^+^ ions and AgNPs can pass via extracellular regions and attach to the various components of biofilm, disrupting bacterial metabolism and compromising essential processes, AgNPs provide benefits in the medical management of biofilm.^97^ In current study, a concentration dependent reduction of biofilm was reported with maximum 82% biofilm inhibition found at 30 µg/ml (Fig. 7). Similar finding was reported by Fernandes *et al*.^95^ inferring strong inhibitory effect against biofilm-producing bacteria by *Cystoseira* produced biogenic AgNPs. Silver nanoparticles produced by the algae *Gelidium corneum* were able to inhibit 50% biofilm formation at 50 μg/mL, similar to the inhibition values of silver@CT and silver@CB.^96^ Comparably, Danaei *et al*.^98^ reported AgNPs synthesized by *Spirogyra* sp. partially prevented the formation of biofilm. Mohanta *et al*.^99^ reported moderate inhibition of biofilm by AgNPs synthesized by the extracts of *B*. *retusa*, *S*. *anacardium*, and *G*. *lanceolarium*. The process proposed involved the liberation of Ag^+^ ions from silver nanoparticles; penetrating the biofilm and piloting the biosorption. These cumulative effects resulted in the killing of bacterial cells and disrupting the production and secretion of EPS (exopolysaccharides). Size is a crucial factor in the interactions of NPs with biofilm, along with shape and surface properties.^100^

Diabetes, which raises glucose levels in the blood and is widely acknowledged as a global health risk, is defined by insufficient insulin production or cellular rejection of insulin.^101^ Constant thirst, hunger, tiredness, frequent urination, unintentional weight loss, and hazy vision are typical symptoms of the condition.^102^ A person's quality of life could be considerably reduced by any number of chronic ailments that could later arise from this circumstance, including neuropathy, cancer, heart disease, stroke, and chronic kidney disease. It has been noted that diabetic wounds affect about 20% of patients globally.^103^ Patients with diabetes have impaired glucose metabolism, which increases the risk of foot and leg ulcers. The lack of coordinated and preventative actions is the reason behind the deterioration in the healing process. In diabetic wounds, between 50–70% of limbs are amputated, and 80% of mortality occurs in developing nations. Although there is an extensive range of diabetic medicines available, medical professionals are worried about the severe adverse reactions and potential toxicity of these drugs when used to treat diabetes.^104^ Furthermore, some patients may become resistant to anti-diabetic treatments because they must be taken regularly. As a result, this dilemma has forced the medical community, particularly pharmaceutical firms and researchers, to look at other options for dealing with the existing issue.^104^ Diabetes research has significantly increased due to this development, and scientists are now concentrating more on the biological applications of silver and compounds based on silver. These substances' many medically valuable qualities, such as their antibacterial, anti-diabetic, and antioxidant activities, are well known.^105^, ^106^ In addition, the application of nanotechnology—more significantly, silver nanoparticles—in treating diabetes has yielded several beneficial effects because of their unique characteristics. In light of biological applications, the THE-AgNPs were analyzed to determine their anti-diabetic potentiality by using the enzymes α-amylase and α-glucosidase. Our results demonstrated that the application of THE-AgNPs significantly inhibited the activities of enzymes α-amylase and α-glucosidase. Additionally, the investigation's studies revealed that the degree of enzymatic inhibition increased with increased AgNPs concentrations (Fig. 8a, Fig. 8b) which corroborated well with the report of Majeed *et al*..^104^ The *Punica granatum* leaf-derived silver nanoparticles (AgNPs) had a similar significant ability to suppress α-amylase.^107^ A dose-dependent pattern of positive connection between the concentration of silver nanoparticles obtained from sea weeds and the inhibition of α-amylase was observed.^108^ The great efficiency of AgNPs synthesized from *Cassia auriculata* in inhibiting α-glucosidase has been reported by Sivakumar.^109^ Likewise, the silver nanoparticles produced from *Ocimum sanctum* (L.) and *O*. *basilicum* showed inhibitory effects on α-glucosidase and noteworthy anti-diabetic qualities.^110^ Therefore, our results align with other studies showing that using THE-AgNPs efficiently inhibits the enzymatic activities of α-amylase and α-glucosidase, implying its possible therapeutic use in treating hyperglycemia in diabetics.

**Fig. 8a:**
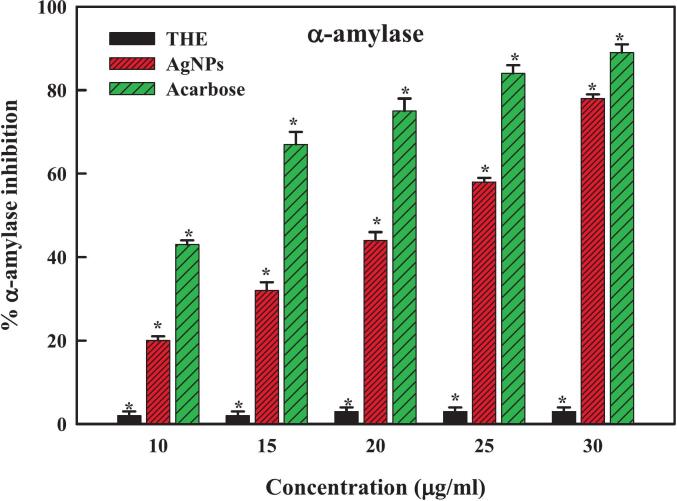
Enzyme α-amylase inhibition activity of THE-AgNPs.

**Fig. 8b:**
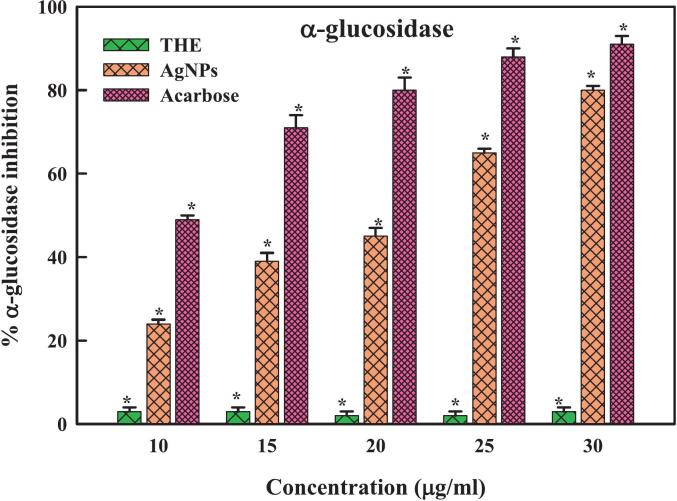
Enzyme α-glucosidase inhibition activity of THE-AgNPs.

**Fig. 9:**
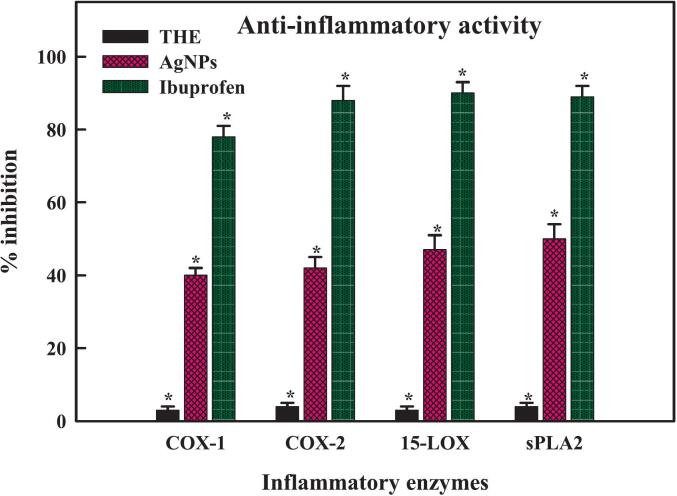
Anti-inflammatory enzymatic inhibition activity of THE-AgNPs.

The body uses inflammation as a mechanical defense mechanism to fight off pathogens, irritants, damaging substances, and damaged cells. Numerous secondary metabolic products and different metallic NPs have exhibited a range of anti-inflammatory properties both in-vitro and in-vivo experimentations. Effective anti-inflammatory properties of flavonoids include their ability to inhibit cyclooxygenase activity against eicosanoids, COX-1 and −2, enzymes phospholipase A2 and lipoxygenases; and lower levels of inflammation-related leukotrienes and prostanoids.^111^ Certain novel and existing medications can treat inflammatory disorders by significantly lowering the clinical signs of inflammatory illnesses.^112^ Metallic nanoparticles have an exceptional capacity to break down bacterial membranes. Consequently, if a microbial infection occurs, the situation can be resolved by rendering a specific medication more soluble. Recent study indicates that nanoparticles are exclusively designed in the prevention and treatment of inflammatory illnesses by the combination of anti-inflammatory chemicals.^112^ Several *in-vitro* assays, including COX-1 and −2, s PLA2, and 15-LOX were employed to evaluate the anti-inflammatory activities of THE-AgNPs. Each of the results of these tests demonstrated practical inhibitory efficacy against every NPs concentration. As seen in Fig. 9, sPLA2 had the most significant inhibitory potential among them (60.3 ± 1.67 %), followed by 15-LOX (54.7 ± 2.01 %), COX-2 (49.43 ± 1.21 %), and COX-1 (45.34 ± 1.12 %), in descending order. The findings demonstrated that THE-AgNPs effectively inhibited the inflammatory processes-related enzymes sPLA2 and 15-LOX. Jan *et al*.^41^ reported similar results of anti-inflammatory efficacy in *Aquilegia pubiflora* assisted ZnO-NPs. The *Delphinium uncinatum* root extract mediated ZnO-NPs reported anti-inflammation potential of 33.2 % (sPLA2), 24.3 % (15-LOX), 17.89 % (COX-2), and 16.53 % (COX-1).^113^

Thus, the nano-formulation of a plant extract improves its biological activity by combining improved transport, synergistic effects, and the distinctive characteristics of nanoparticles, according to published scientific studies. Due to low absorption, low stability, and low bioavailability, a pure plant extract by itself frequently has limited efficacy; these issues are resolved by nano-formulation.^114^ The mechanistic reasons for enhanced activity are mentioned as follows:

1. Increased surface area and solubility: Compared to bulk materials, nanoparticles (NPs) have a substantially higher surface-area-to-volume ratio.
a) Improved dissolution: Nano-formulation significantly boosts the effective surface area of poorly water-soluble or hydrophobic phytochemicals, which are frequently found in plant extracts. As a result, most active phytochemicals can dissolve more quickly and completely, increasing their availability for biological action.^115^
b) Better absorption: The nano-formulation can more readily pass through biological barriers including mucosal layers and cell membranes because of its lower particle size. Compared to the greater molecular size of compounds in a crude extract, this results in improved uptake and transportation throughout the body.^116^
2. Synergistic effects: Silver nanoparticles (AgNPs) and plant extracts frequently work in concert to produce a synergistic effect, in which the total activity of the two substances is higher than the sum of their separate effects.^114^
a) Improved antibacterial capabilities: AgNPs have the ability to harm bacterial cell membranes and interfere with the ETC, which results in leakage and cell death. When combined with phytocompounds, which also have antibacterial qualities, this impact is enhanced. This two-pronged technique works better than either one by itself.
b) Combined mechanisms: In addition to stabilising the nanoparticles and avoiding aggregation, the phytochemicals may also function as reducing and capping agents during the AgNPs production. Both the medicinal qualities of the phytochemicals and the impacts of the metallic nanoparticles are carried in one system by the resulting “phytoconjugated” nanoparticles.^117^
3. Controlled and targeted release: Unlike crude extracts alone, nano-formulations enable the exact and regulated distribution of active chemicals.
a) Passive targeting: Nanoparticles can passively gather in specific tissues or organs through passive targeting. Nanoparticles can be used in cancer treatment, for instance, by taking advantage of the EPR effect, which occurs when tumours accumulate because of leaky vasculature.
b) Persistent distribution: The active ingredients may be gradually released over a long time using the nanocarrier system, thereby reducing the possibility of adverse effects by enabling lower, less frequent doses and a more constant therapeutic impact.^118^
4. Enhanced security and stability
a) Protection against deterioration: Environmental elements such as light, heat, and pH variations can cause herbal medications and the phytochemicals that make them active to break down and lose their effectiveness. The extract is shielded from the severe environment of the digestive tract and early enzyme breakdown by being encapsulated in a nanocarrier.
b) Increased shelf-life: Nano-formulations make herbal medicines more stable and durable by shielding their active ingredients from environmental deterioration, which prolongs their duration of storage.^116^
5. Increased absorption by cells: Nanoparticles can infiltrate cells more efficiently than bigger unformulated molecules because of their small size. Studies on a variety of therapeutic substances have shown that a lower dosage of the nano-formulation can result in a more substantial and quicker biological response due to the improved cellular absorption.^119^

Many industrial sectors use organic dyes, including cosmetics, ceramics, plastic, paper, textiles, and pharmaceuticals. As they are mutagenic and carcinogenic, introducing them into wastewater poses significant hazards to environmental and human health.^120^ Because of the dyes' stable aromatic nature, it has been discovered that the conventional techniques employed to combat them are ineffective. Given these issues, the field of nanocatalysis—this employs metal nanoparticles in the breakdown of hazardous dyes—is up-and-coming.^121^ The catalytic potential of THE-AgNPs was examined experimentally by employing the dye molecules of methyl orange and safranin O. The heterocyclic dye safranin contains azine group and the former’s presence in wastewater can negatively affect aquatic environments. The distinctive UV–Vis peak of safranin O is visible at 520 nm. Using NaBH_4_ as a catalyst and THE-AgNPs as test material, the catalytic activity of safranin O was experimentally observed by spectroscopic analysis. The reducing power of NaBH_4_ was found to be low in the absence of AgNPs (blank test). The dye SO degraded by adding varying amounts of THE-AgNPs to the NaBH_4_ and SO solution mixture, leading to successively reduction in the dye absorption intensity (Fig. 10a). The reddish hue of the dye SO was converted to colorless hydrazine derivatives with % degradation calculated as 95%. The reaction followed pseudo first order dynamics displayed on a kinetically time-dependent graph, and the constant K was determined as 0.0332 min^−1^. The reduction dynamics corroborated well with the studies of Muhmood *et al*.^120^; Farooq *et al*.^121^; Vijayan *et al*.^122^; Barman *et al*.^123^; and Mahmood *et al*..^124^ Similarly, an azo dye called methyl orange (MO) was degraded by the THE-AgNPs inferring broad spectrum catalytic activity. A transition of the azo group causes methyl orange to typically be visible at a wavelength of 464 nm. Fig. 10b also shows the UV–visible spectra of MO. Fig. 10b depicts the methyl orange degradation following the addition of NaBH_4_ and THE-AgNPs. It is evident that even with the addition of NaBH_4_, the MO solution still exhibits a similar peak at 464 nm, indicating that the −N=N- moiety still predominates and only slightly reduces the absorbance value. The spectrophotometric measurement of the methyl orange degradation trend following the introduction of both THE-AgNPs and NaBH_4_ was confirmed by a steady decline in the intensity of absorptive peak. Furthermore, a substantially slower reaction rate was observed in the MO degradation process using NaBH_4_ in the absence of a catalyst. The dye degradation trend and kinetically plotted graph with THE-AgNPs acting as a catalyst are shown in Fig. 10a, Fig. 10b. The dye decomposition percentage was close to 96%. The rate constant K value was determined to be 0.0411 min^−1^. This is because when the amount of AgNPs in the catalyst rises, so does its active site's surface area resulting in the gradual rise in the pace of the reaction. This demonstrated that dye MO was effectively degraded by THE-AgNPs through excellent photocatalytic activity. The reduction dynamics corroborated well with the studies of Farooq *et al*.^121^; Barman *et al*.^123^; Edison *et al*.^125^; Joseph *et al*.^126^; and Hashemi *et al*..^127^ A plethora of publications are available regarding the catalytic behavior of AgNPs in opposition to the breakdown of perilous dyes generated chemically and biologically.^121^, ^128^, ^129^, ^130^ These investigations conclude that smaller particles typically exhibit superior catalytic activity, reducing the time needed for degradation. In turn, because of the greater surface area that may be achieved, the accessibility of catalysts also raises the efficacy of catalytic activity. The following illustrates the general reaction that occurs during photocatalysis using sunlight (Fig. 11). The reusability of THE-AgNPs were found to be 95% upto 7 cycles (Fig. 12).

$$AgNPs+hv\to AgNPs\;(e_{CB}^{-}+\;h_{VB}^{+})$$

$$AgNPs\;\left(huv\right)+H_{2}O\to \;{Ag}^{0}+\;H^{.}+\;{OH}^{-}$$

$$O_{2}^{-}+\;H^{+}\to \;{HO}_{2}$$

$$Dye+\;{OH}^{-}\to Degraded\;products$$

$$Dye+\;e_{CB}^{-}\to Reduced\;products$$

$$Dye+\;h_{VB}^{+}\to Oxidized\;products$$

**Fig. 10a:**
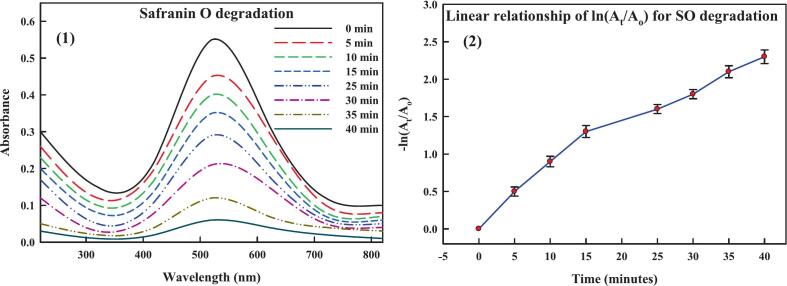
(1) Degradation of dye safranin O (2) Reaction kinetics of dye versus time.

**Fig. 10b:**
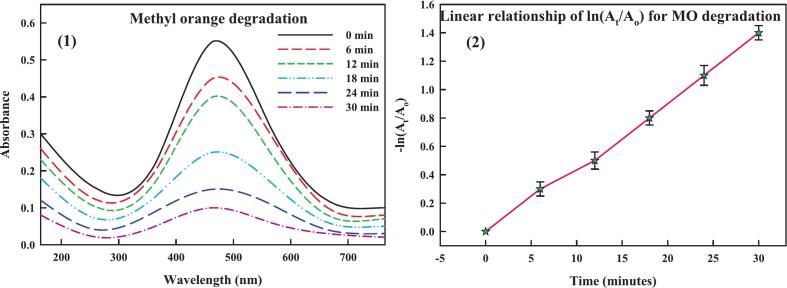
(1) Degradation of dye methyl orange (2) Reaction kinetics of dye versus time. (For interpretation of the references to color in this figure legend, the reader is referred to the web version of this article.)

**Fig. 11:**
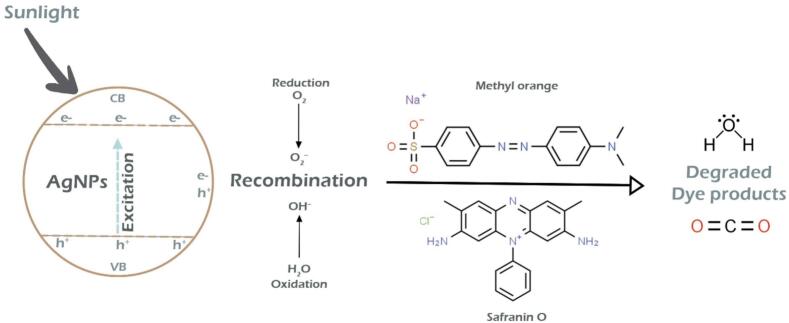
Mechanism of dye degradation assisted by AgNPs.

**Fig. 12:**
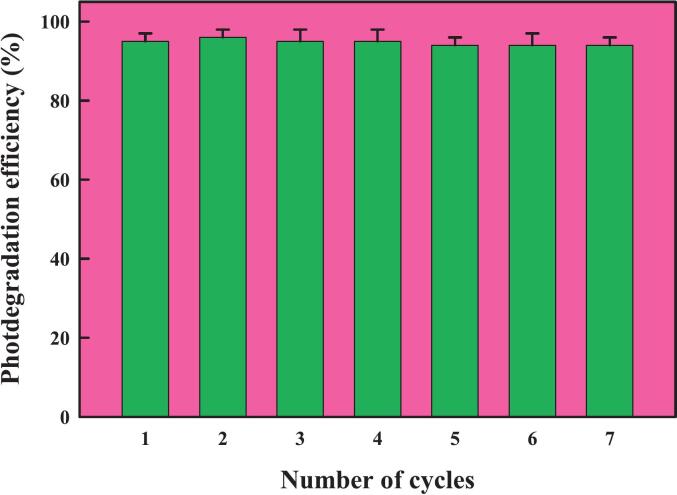
Reusability of THE-AgNPs in the photodegradation of dyes.

### 3.2 Limitations

Even though green nanoparticle manufacturing has numerous benefits, there are still some restrictions that should be taken into account and investigated, particularly with regard to the general physiochemical characteristics of the produced AgNPs. First of all, it is not possible to produce AgNPs on a big scale while utilizing all of the natural resources that are accessible. The overall physiochemical characteristics of the synthesized AgNPs will be impacted by a number of environmental factors, including availability, weather, time restrictions, and overall plant yield. Second, the specific functioning circuitry during the green manufacturing of nanoparticles is not well understood. Although the general contribution of plant sources to the formation of AgNPs is known, the specifics of the reaction kinetics and processes are yet unknown. More significantly, the general shape of the nanoparticles made from various plant sources can change significantly. The total chemical characteristics of the green produced nanoparticles are significantly influenced by these physical parameters. In order to create highly consistent nanoparticles for their potential use as multivariate potencies in a variety of disciplines, more study is therefore needed.

### 3.3 Future perspectives

The scientific community has made a number of coordinated efforts over the last decades to advance the synthesis of silver nanoparticles using plant-based extracts. Green synthesis may be readily scalable for big-ticket commercial synthesis of the nanoparticles as it is affordable and uses eco-friendly techniques. Green synthesis-derived AgNPs have the potential to be employed in a variety of medicinal applications, particularly as antiviral, antifungal, antibacterial, and anticancer drugs in specialized drug medications.^131^ The nanoparticles disturb the activity of cancers, viral, bacterial cells, and fungal elements, sans interference with the regular functioning of human cells. Still, more effort is needed to address a few issues with green synthesis techniques. For example, the raw materials are only available in a certain area, the AgNPs have irregular sizes, and extraction methods are labour-intensive. Furthermore, the presence of an array of phytochemicals in plant extracts makes it very difficult to track how they interact with the produced nanoparticles. Additionally, a number of studies show that AgNPs can be effectively used as antimicrobials and anticancer agents in a variety of laboratory settings; however, these are still small-scale investigations, and further research is needed to determine how they can be used on a wider scale. The effects of green produced AgNPs in a natural environment distinct from the lab still require investigations due to the laboratory settings accessible during the synthesis process and their usage as a therapeutic agent. Lucid and comprehensive researches are required to gain a deeper understanding of plant extracts' chemical constitution and concentration for AgNPs production. The compounds in the plant-based extracts have an impact on the stability and structural integrity of the synthesized AgNPs.^132^ The majority of research show that the dimensions of the nanoparticles decreases as the capping activity increases.^133^ Consequently, additional researches are warranted to comprehend the interactions between AgNPs and phytochemicals. Additionally, the quantity of AgNPs rises with higher levels of the plant extract.^134^ This is only true up to a point, after which agglomeration results from the production of massive nanoparticles.^134^

## 4 Conclusion

It has been discovered that AgNPs have remarkable qualities with several uses, especially in the medical and environmental fields. Due to their synergistic effects, silver combined with other metals can enhance their qualities. The production of green AgNPs nanoparticles from *Thalassia hemprichii* leaf blades extract has been described in this study for the very first time. No hazardous ingredients are used in green synthesis. The output of multigrain agglomerates made up of incredibly tiny crystallites was revealed by the SEM investigation. Even after the dye degradations, the produced spherical AgNPs didn’t demonstrate any discernible alterations. The antibacterial potential was also evaluated against bacterial strains that demonstrated antibacterial activity action, possibly related to the production of ROS and ensuing membrane damage. The anti-diabetic, anti-inflammatory, antioxidant properties of AgNPs can be of great relevance in pharmaceutics and nanomedicines. The produced NPs' catalytic activity remained relatively high until the seventh cycle of the recycling experiment, indicating it as a potential photocatalyst. The AgNPs may be investigated for creating nanocomposite structures in the future due to their morphological characteristics and notable zeta potential in various medicinal uses and other environmental uses.

## Ethical statement

Every usage of microorganisms in this research endeavour was carried out in compliance with accepted ethical standards, guaranteeing appropriate handling, containment, and disposal. Every possible danger to people, animals, and the environment was reduced. In order to maintain the highest standards of safety and scientific integrity, all procedures adhered to institutional biosafety regulations.

## CRediT authorship contribution statement

**Shibin Eranhottu:** Writing – review & editing, Investigation, Formal analysis. **Tijo Cherian:** Writing – review & editing, Writing – original draft, Software, Methodology, Investigation, Formal analysis, Conceptualization. **R. Mohanraju:** Validation, Supervision, Resources, Project administration, Funding acquisition, Data curation. **N. Sharmila Devi:** Writing – review & editing. **Jayalakshmi Sanal:** Writing – review & editing. **Lincy Sara Varghese:** Writing – review & editing. **P. Ambili:** Writing – review & editing. **Mini Thomas:** Writing – review & editing. **Fahmeeda Parveen P.S.:** Writing – review & editing. **Willie J.G.M. Peijnenburg:** Writing – review & editing.

## Declaration of competing interest

The authors declare that they have no known competing financial interests or personal relationships that could have appeared to influence the work reported in this paper.

## Data Availability

Data has been made available in the manuscript.
