# Supplementary material for: Eco-friendly synthesis of silver nanoparticles from the leaf blades extract of seagrass Thalassia hemprichii: Multifunctional agents for biomedical and environmental applications
Source: J Genet Eng Biotechnol. 2025 Oct 22;23(4):100600. doi: 10.1016/j.jgeb.2025.100600 (PMC12590284; doi:10.1016/j.jgeb.2025.100600)
Supplement: Supplementary Data 1 [file mmc1.docx]

**3. Instrumentation details-**

| **S. No** | **Reagents/Instrumentation** | **Manufacturer/Model specifications** |
| --- | --- | --- |
| 1. | FT-IR | FT-IR spectrometer Spectrum Two, Perkin Elmer Life and Analytical Sciences, Waltham, MA, USA [THE and THE-AgNPs were separately mixed with spectroscopic grade of KBr in a ratio of 1:100, followed by the recording of spectra at a resolution of 4 cm^−1^]. |
| 2. | UV-visible spectroscopy | Cintra 101, GBC Scientific Equipment Ltd., Braeside, Australia. Range 250–700 nm. |
| 3. | Electron microscopy (TEM, SEM) | JEOL, Tokyo, Japan  [SEM: 20 kV (JEOL Ltd., Tokyo, Japan). Fine powder of THE-AgNPs was placed on the carbon tape, followed by gold coating using vacuum sputter.]  [TEM: JEOL, Tokyo, Japan) at an accelerating voltage of 200 kV. Samples were prepared by dispensing 10 μL THE-AgNPs on to the Cu grid, and the liquid was vacuum dried (80^°^C, 6 h]. |
| 4. | Zeta potential | Zeta Sizer Nano ZS-90 Malvern Instruments, Malvern, UK |

**2.5 Anti-diabetic activity of AgNPs**

**2.5.1 α-amylase inhibition assay.** The anti-diabetic potential of AgNPs was assessed using the α-amylase inhibition assay (Bibi *et al*., 2011). In a sterile microtitre plate, phosphate buffer (15 µl; pH 6.8) and α-amylase enzyme (25 µl; 0.14 U/ml) were added. Following, 40 µl starch solution and 10 µl AgNPs (concentrations 10-30 µg/ml) were added together, and the mixture was incubated at 50^°^C for 30 minutes. After the incubation period, the mixture was mixed with iodine reagent (90 µl; 5 mM of each potassium iodide and iodine) and 1 M HCl (20 µl). Three reaction controls were taken in parallel: a blank solution without AgNPs and enzyme, a negative control (solution lacking test sample), and a positive control (acarbose). At 595 nm, the OD values were determined and the following equation (Eq. 1) was used to calculate the % enzyme inhibition:

% Enzyme inhibition = OD(s)-(OD (n))/(OD (b))×100 ------- (Eq. 1)


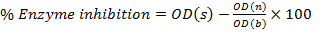


where OD (b) indicates blank, OD (s) indicates absorbance value of test sample, and OD (n) indicates negative control.

**2.5.2 In vitro α‑glucosidase inhibition assay.** The ability of AgNPs to suppress the activity of the enzyme α-glucosidase was determined using a slightly modified version of Sheliya *et al*. (2016) methodology. The reaction mixture consisted of acarbose, AgNPs (10-30 μg/ml), and α-glucosidase [7.5 µl; stock solution (0.5 U/ml) in sodium phosphate buffer (pH 6.9; 20 mmol/l)] incubated for 15 minutes at 37^°^C. Additionally, 100μl PNPG (p-nitrophenyl-α-glucopyranoside) was added, and the mixture was incubated for 10 minutes at 37^°^C. At last, the process was bunged by introducing 100μl sodium carbonate (Na_2_CO_3_; 0.1 M). At 405 nm, the absorbance values were measured with acarbose and PNPG + α-glucosidase as the reference and control solutions, respectively.

% inhibition = (control OD-test OD)/(control OD)×100 ------- (Eq. 2)


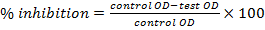


**2.6 Antioxidant activities of AgNPs**

**2.6.1 FRSA (Free radical scavenging assay).** The efficacy of AgNPs to scavenge free radicals was examined using the DPPH (2,2-diphenyl 1-picrylhydrazyl) test (Fatima *et al*., 2015). In brief, 190 µL of DPPH reagent and 10 µl AgNPs were added and incubated for 30 minutes at 37^°^C. The ascorbic acid positive control was run in parallel; the absorbance (OD 515 nm) was measured, and the free radical scavenging activity was assessed using the following equation (Eq. 3):

% Scavenging = 1-AE/AD×100------- (Eq. 3)

where AE= absorbance of test sample solution and AD= negative control.

**2.6.2 TAC (Total antioxidant capacity).** Using the phosphomolybdenum technique, the total antioxidant activity of AgNPs was measured (Zafar *et al*., 2016). In summary incubating 900 µl of phosphomolybdenum reagent [sodium phosphate (28 mM), ammonium molybdate (4 mM), and sulphuric acid (0.6 M)] was mixed with 100 µl AgNPs followed by incubation for 90 minutes at 95^°^C. The absorbance of reaction mixture (200 µl) was measured at 695 nm and the amount of ascorbic acid equivalents to ascorbic acid per mg of test sample (mg AAE/mg) was used to determine the antioxidant activity. Ascorbic acid served as positive control.

**2.6.3 TRP (Total reducing power).** Using an experiment based on potassium ferricyanide, the overall reducing power of AgNPs was examined (Zafar *et al*., 2016). The total reducing power was measured using iron as a reducing agent. The reaction mixture, consisting of 40 µl AgNPs, 1% aqueous potassium ferricyanide, and 400 µl phosphate buffer (pH 6.6, 0.2 mol/l), was incubated for 20 minutes at 45^°^C. Following incubation, aqueous trichloroacetic acid (10%; 400 µl) was added, and the mixture was subjected to centrifugation at 3000 rpm for 10 minutes. The resultant supernatant (500 µl) was combined with the same amounts of sterile distilled water and 100 µl of aqueous FeCl_3_ (0.1%). Following the absorbance measurement at 630 nm, the outcomes were reported as mg AAE/mg. The reaction controls were conducted in parallel using DMSO as the blank and ascorbic acid as the positive control.

**2.6.4 ABTS antioxidant assay.** The ABTS test was carried out according to Shah *et al*. (2019) guidelines. The mixture containing potassium persulphate (2.5 mM) and ABTS salt (7mM) in equal amounts was stored in the dark for 14–16 hours. The absorbance at 734 nm was measured and normalized to 0.7 before adding AgNPs. After that, the reaction mixture was mixed with the various concentrations of AgNPs, and it was left to incubate at room temperature for 15 minutes under dark. The Trolox C equivalent antioxidant capacity (TEAC) was used to quantify the activity after the OD at 734 nm was measured.

**2.7 Anti-inflammatory potential of AgNPs**

**2.7.1 COX-1 and COX-2 inhibitory activities**

COX-1 and COX-2 were used to examine the inhibitory potential of AgNPs (Jan *et* *al*., 2021). Ibuprofen (10 mM) Arachidonic acid (1.1 mM) were taken as positive control and substrate. Both COXs peroxidase constituents were calculated by following manufacturer's instruction kit. After brief incubation of 5 minutes, the absorbance was read at 590 nm for the quantification of N,N,N/,N/-tetramethyl-p-phenylene diamine.

**2.7.2 Inhibitory activity of AgNPs against 15-LOX**

The inhibitory activity of AgNPs against 15-LOX was examined (Jan *et* *al*., 2021). Arachidonic acid (10M) and NDGA (100M; nordihydroguaiaretic acid) served as substrate and positive control, respectively. Standard (soy 15-lipooxygenase) was used in the evaluation of hydroperoxides concentration formed as a result of lipo-oxygenation in Tris-HCl buffer (10mM; pH 7.4). The reaction mixture was incubated for 5 minutes and the absorbance was read at 940 nm. Values of absorbance were measured: value A (post 15 min. of substrate incubation) and value B (5 min. inhibitor-enzyme incubation followed by 5-min. chromogen incubation).

**2.7.3 Inhibitory potential of AgNPs against secretory phospholipase A2 (sPLA2)**

The assessment of inhibitory activity of AgNPs against sPLA2 was ascertained by following the method of Jan *et* *al*. (2021). The compound, thiotheramide-PC (100mM) was taken as substrate and diheptanoyl thio-PC (1.44mM) served as positive control. The free thiols species, released by the breakdown of diheptanoyl thio-PC ester, were quantified by using DTNB (5-50-dithio-bis-butyl) and the absorbance was read at 420 nm. The value of % inhibition was calculated by following formula:

% inhibition=(IA-Inhibitor)/IA×100

**2.8 Antibacterial activity of AgNPs**

**2.8.1 Growth and preservation of pathogenic strains**

*Staphylococcus* *aureus* and *Escherichia* *coli* were the two bacterial pathogens that were routinely subcultured on Mueller Hinton Agar and kept as culture stock [10% glycerol stocks (-20^°^C), slants/stabs (4^°^C)].

**2.8.2 MIC (Minimal Inhibitory Concentration) of AgNPs**

The broth microdilution method (Baker *et al*., 1991; Cherian *et al*., 2022) was used to determine the MIC. Silver nanoparticles at varying concentrations (10-30 µg/ml) were evaluated and kept at 37^°^C for 24 hours with control tubes. At 620 nm, the absorbance values were recorded.

**2.8.3 MBC (Minimal Bactericidal Concentration) of AgNPs**

From MIC tubes, aliquots (20 µl) were seeded, cultivated on nutrient agar media, and incubated at 37^°^C for 24 hours in order to determine MBC (Hausdorfer *et al*., 1998; Cherian *et al*., 2020).

**2.8.4 Antibacterial activity of AgNPs**

The agar well diffusion method (Magaldi *et al*., 2004; Valgas *et al*., 2007; Cherian *et al*., 2022) was used to measure the bactericidal activity of AgNPs against pathogens. The 0.1 ml culture (2×10^8^ CFU/ml) was evenly distributed over MHA media. Variable concentrations of silver nanoparticles (10-30 µg/ml) were added to equal-sized wells, and the latter were incubated for 24 hours at 37^°^C. The ZOI (zone of inhibition) was measured using Antibiotic Zone Scale (HiMedia, India), and cefixime (an antibiotic) served as the positive control.

**2.8.5 Anti-biofilm potential of AgNPs**

Crystal violet (CV) assay was used to assess the anti-biofilm efficacy of AgNPs (Cherian *et al*., 2022). The 96-well microtitre plates were seeded with starter cultures (cell density ~10^8^ cells/ml; 100 µl) cultivated overnight in nutrient broth. Variable concentrations of silver nanoparticles (10-30 µg/ml; 100 µl) in combination with a control group of untreated cells were added to each well, and the mixture was incubated for 24 hours at 37^°^C. Three rounds of washings using 1:1 autoclaved distilled water + phosphate buffer saline (PBS) were performed on the residual loosely bound cells. After adding the CV solution (0.25%, 200 µl), the mixture was incubated for 30 minutes at 37^°^C. Autoclaved distilled water mixed with PBS was used to wash the unbound CV. After dissolving the bound CV-bacterial cells in 250 µl of 95% ethyl alcohol, the absorbance at 620 nm was measured.

**2.9 AgNPs assisted dye degradation studies**

The degradation of dyes (Methyl orange and Safranin O) was examined using AgNPs as nanocatalysts. As monitor wavelengths, the dyes MO and SO exhibit absorbance maxima at 464 nm and 520 nm, respectively. The dye solutions were agitated for 20 minutes in the dark after adding the proper quantity of photocatalyst (AgNPs) to achieve adsorption/desorption equilibrium. After shaking the mixture manually, the absorption spectra were recorded at room temperature. The absorbance change over time was used to quantify the catalytic degradation. The % degradation was computed by the following equation:

D=(C_o_-C_t_)/C_o_×100

where C_o_ and C_t_ = dye concentrations at time 0 min and t min, respectively.

Fig. A1 Actual kinetic plots (ln(C/C₀) vs. time) for dye Methyl orange

Fig. A2 Actual kinetic plots (ln(C/C₀) vs. time) for dye Safranin O
